# How much of protein sequence space has been explored by life on Earth?

**DOI:** 10.1098/rsif.2008.0085

**Published:** 2008-04-15

**Authors:** David T.F Dryden, Andrew R Thomson, John H White

**Affiliations:** School of Chemistry, University of EdinburghThe King's Buildings, Edinburgh EH9 3JJ, UK

**Keywords:** protein sequence, evolution, contingency

## Abstract

We suggest that the vastness of protein sequence space is actually completely explorable during the populating of the Earth by life by considering upper and lower limits for the number of organisms, genome size, mutation rate and the number of functionally distinct classes of amino acids. We conclude that rather than life having explored only an infinitesimally small part of sequence space in the last 4 Gyr, it is instead quite plausible for all of functional protein sequence space to have been explored and that furthermore, at the molecular level, there is no role for contingency.

## 1. Introduction

Two assumptions are generally made when considering the molecular evolution of functional proteins during the history of life on Earth. Firstly, the size of protein sequence space, i.e. the number of possible amino acid sequences, is astronomically large and, secondly, that only an infinitesimally small portion has been explored during the course of life on Earth (e.g. Salisbury 1969; Maynard Smith 1970; Mandecki 1998; Luisi 2003; Carrier 2004; de Duve 2005). Luisi and Chiarabelli have termed the unexplored part of sequence space as containing the ‘never born proteins’ (Luisi *et al*. 2006; Chiarabelli & de Lucrezia 2007). We wish to discuss these two assumptions by estimating how much of this space could have been explored since the origin of life some 4 Gyr ago. As will be described below, others have concluded that the first assumption is incorrect and we agree with this conclusion. However, we also conclude that the second assumption is incorrect and calculate that most of the sequence space may have been explored.

Before turning to a discussion of the second assumption, we wish to summarize information showing the first assumption, namely that the sequence space is vast, to be false. A typical estimate of the size of sequence space is 20^100^ (approx. 10^130^) for a protein of 100 amino acids in which any of the normally occurring 20 amino acids can be found. This number is indeed gigantic but it is likely to be a significant overestimate of the size of protein sequence space. For example, Dill and colleagues used simple theoretical models to suggest (Lau & Dill 1990; Chan & Dill 1991; Dill 1999), and experimental or computational variation of protein sequence provides ample evidence (Cordes *et al*. 1996; Riddle *et al*. 1997; Plaxco *et al*. 1998; Larson *et al*. 2002; Guo *et al*. 2004; Doi *et al*. 2005), that the actual identity of most of the amino acids in a protein is irrelevant. An example in nature could be the prokaryotic DNA methyltransferases which each contain a target recognition domain (TRD) of approximately 150 amino acids that recognizes specific DNA sequences usually of 3–6 bp in length, and a conserved catalytic domain. The thousands of known TRD sequences show negligible amino acid sequence conservation despite the rather limited number of nucleotide sequences they are required to recognize (e.g. Sturrock & Dryden 1997; O'Neill *et al*. 1998; Bujnicki 2001; Roberts *et al*. 2007). As an extreme method to reduce the size of sequence space, Dill (1999) suggested that only two types of amino acid were needed to form a protein structure, hydrophilic and hydrophobic, and that furthermore it was critical to define only the surface of the protein. These two suggestions reduce the size of sequence space to 2^100^ and 2^33^, respectively (i.e. approx. 10^30^ and approx. 10^10^). It is noteworthy that recent coarse-grained ‘tube’ models go even further and remove all atomic information leaving only a potential energy function for interaction with other parts of the tube. Despite the extreme coarse graining of this model, recognizable ‘protein’ structures can still be found (Banavar *et al*. 2006). Although this may appear to go against Anfinsen's dogma that a protein structure is determined by its amino acid sequence (Anfinsen 1973), it is really only a case of an extreme reduction in the size of the amino acid ‘alphabet’. The tube structures obtained are rather similar to the short folded segments adopted by sequences apparently conserved since the last universal ancestor (Sobolevsky & Trifonov 2006). The assumption that a protein chain needs to be at least 100 amino acids in length also rather inflates the size of sequence space when it is known that many proteins are modular and contain domains of as few as approximately 50 amino acids thereby reducing the space to 20^50^ or approximately 10^65^ (e.g. Sobolevsky & Trifonov 2006). The conclusion from all of these coarse-graining approaches is that a reduced alphabet of amino acids is quite capable of producing all protein folds (approx. a few thousand discrete folds; Denton 2008) and providing a scaffold capable of supporting all protein functions (we will ignore the space of natively unfolded proteins for this current discussion but since such proteins usually fold upon performing their function, the distinction is not important for our purposes; Dyson & Wright 2005). The phase space of function may be some orders of magnitude greater than the size of the folding space as metagenomics projects are revealing increasing numbers of unknown protein families as adjudged by the number of novel protein sequences (Raes *et al*. 2007). However, it is not clear that new folds are present as a conserved fold, such as the TIM barrel, is capable of displaying many functions (Nagano *et al*. 2002).

To further support this idea of a reduced alphabet of amino acids, there are also very plausible suggestions that the original amino acid repertoire consisted of only four or five amino acids like those found in the Miller–Urey experiments and the Murchison meteorite (Miller *et al*. 1976), and that the genetic code was initially limited to these few amino acids that still predominate in proteins to the current day (e.g. Trifonov 2000; Brooks *et al*. 2002; Ikehara 2002). Proteins with reduced amino acid repertoires can fold and function successfully (e.g. Cordes *et al*. 1996; Riddle *et al*. 1997; Plaxco *et al*. 1998; Guo *et al*. 2004; Doi *et al*. 2005; López de la Osa *et al*. 2007).

Figure 1 shows the number of possible sequences as a function of the number of different amino acids (or classes of amino acids, 1–20) and the length of the functionally important amino acid chain (33, 50 or 100). It highlights the drastic reduction in the size of sequence space if one limits the number of available amino acid types to less than the 20 usually found today, a limitation that appears to be justified experimentally.

## 2. Results

We now wish to consider the second assumption commonly made about protein sequence space: that only an infinitesimal fraction has been explored by life on Earth. To examine how much of sequence space could have been explored, it is simplest to make upper and lower limit estimates for the number of unique amino acid sequences produced since the origin of life using some liberal assumptions. Considering the upper limit, it is clear that bacteria dominate the planet in terms of the product of the number of cells (10^30^; Whitman *et al*. 1998) multiplied by the number of genes in each genome (10^4^, a small overestimate). Let us assume that every single gene in this total of 10^34^ is unique and that evolution has been working on these genes for 4 Gyr completely changing each gene to some other unique, new gene every single year. This gives an extreme upper limit of 4×10^43^ different amino acid sequences explored since the origin of life. The contribution to this number of sequences by viral and eukaryotic genomes is difficult to estimate but it is very unlikely to be orders of magnitude greater than the 4×10^43^ sequences from bacteria. If their contribution is similar or smaller, then it can be ignored in our rough calculation. For comparison with our calculation, Mandecki (1998) gave a limit of 10^50^ protein sequences since the origin of life. A lower limit to the number of sequences explored is more difficult to estimate but it has been estimated that there are 10^9^ different bacterial species on Earth (Whitman *et al*. 1998; Medini *et al*. 2005; Simonson *et al*. 2005). If we assume that each species has a unique complement of 10^3^ sequences (an underestimate) and that only one sequence has changed per species per generation (a reasonable estimate based upon analysis of mutation rates in bacteria; Perfeito *et al*. 2007), and that the generation time is 1 year (a considerable underestimate for many modern bacteria (Ochman *et al*. 1999), but perhaps reasonable for an ancient organism or one growing slowly in a poor environment), then we arrive at a figure of 4×10^21^ different protein sequences tested since the origin of life.

These two limits are shown in figure 1. Although the oft-quoted 20^100^ (approx. 10^130^) size of sequence space is far above these limits, the other more plausible estimates for the size of sequence space, particularly with limited amino acid diversity or reduced length, are near to or within these two limits. Considering the upper limit, all sequences containing 20, 8 and 3 types of amino acids have been explored if the chains are 33, 50 and 100 amino acids in length, respectively. Considering the lower limit, then virtually all chains of length 33 and 50 amino acids containing five or three types of amino acid, respectively, could have been explored. (The exploration of longer chains of 100 amino acids with only two types of residue is obviously much less complete but it is not a negligible fraction of the total.) Therefore it is entirely feasible that for all practical (i.e. functional and structural) purposes, protein sequence space has been fully explored during the course of evolution of life on Earth (perhaps even before the appearance of eukaryotes).

## 3. Discussion

Protein sequence space is often viewed as a limitless desert of maladjusted sequences with only a few oases of working sequences linked by narrow pathways (Axe 2000, 2004). The navigation over this space by natural selection is difficult and could take many different routes thus resulting in organisms with largely different protein compositions. This idea of contingency, if taken at the level of species, led Gould to suggest that if one was to rerun the ‘tape of life’ then evolution would take a totally different path and we, as a species, would only appear as a highly improbable accident (Gould 1991; Luisi 2003; de Duve 2007*a*,*b*). However, if there is any merit to our simple calculation then protein sequence analysis provides no support for the idea of contingency at a molecular level and it provides strong support for the ideas of convergence (Conway Morris 2000, 2004; Dawkins 2005; Vermeij 2006; de Duve 2007*a*,*b*). If one was to rerun the tape, then the protein composition of organisms would be similar. Our calculation removes the almost impossibly unrealistic pressure on natural selection to navigate through protein sequence space avoiding the vast number of functionless sequences by simply indicating that most sequences have been tried are useful in some way, and that there are many possible routes to obtain proteins with desirable functions (Nagano *et al*. 2002; Anantharaman *et al*. 2003; Holliday *et al*. 2007).

Finally, we conclude that the number 20^100^ and similar large numbers (e.g. Salisbury 1969; Maynard Smith 1970; Mandecki 1998; Luisi 2003; Carrier 2004; de Duve 2005) are simply ‘straw men’ advanced to initiate discussion in the same spirit as the ‘Levinthal paradox’ of protein folding rates (Levinthal 1969; Zwanzig *et al*. 1992). 20^100^ is now no more useful than the approximate 2×10^1 834 097^ books present in Borges' (1999) fantastical ‘Library of Babel’ and has no connection with the real world of amino acids and proteins. Hence, we hope that our calculation will also rule out any possible use of this big numbers ‘game’ to provide justification for postulating divine intervention (Bradley 2004; Dembski 2004).

## Figures and Tables

**Figure 1:**
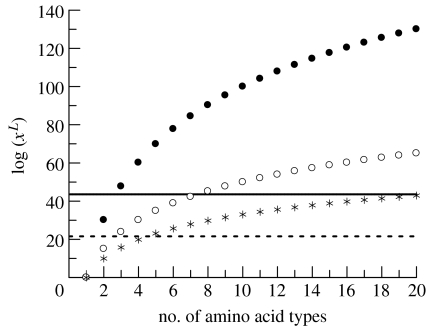
The size of protein sequence space log(*x*^*L*^) as a function of the size of the amino acid alphabet (i.e. the number of different types of amino acids, *x*) for proteins containing 33 (asterisks), 50 (open circles) or 100 (filled circles) amino acids (length *L*). The horizontal lines represent estimates of the maximum (solid line) and minimum (dashed line) number of sequences explored during the 4 Gyr since the origin of life on Earth.
